# P316: Old peoples’ condom use and perception: a big challenge to mitigation of HIV/AIDS in high risk urban slums in Nigeria

**DOI:** 10.1186/2047-2994-2-S1-P316

**Published:** 2013-06-20

**Authors:** K Odor, R Opara, N Iwuji

**Affiliations:** 1 Health Promotion, University of Ibadan, Nigeria, Ibadan, Nigeria; 2 Aged Care, Gertrude Abbot Nursing Home, Surry Hills, Australia; 3 Immunization/Research, Olive Hospital and Maternity/Sure health org, Aba, Nigeria

## Background/significance

As HIV/AIDS continues to pose a public health challenge in Africa, the pandemic cut-across borders. It affects all the age groups including old persons, despite engagement in risky sexual activities which increases HIV/AIDS infection. However, limited attention is paid to this sub-group in mitigating the pandemic. This study therefore examined condom-use and perceived HIV/AIDS infection among old people in Nigeria.

## Methodology

The study was cross-sectional in design. A multi-stage sampling procedure was used to select 400-geriatrics. Pre-tested questionnaire developed, using information obtained from 10 Focus Group Discussion (FGD), was used to collect information. FGD data were analyzed thematically, while questionnaire data were analyzed using descriptive and statistically.

## Findings

Twenty-five percent of the participants had extra-marital sex since they attained elderly age. However, among this subgroup that had extra-marital sex, few (6.8%) used a condom. More males (5.3%) than females (1.5%) used condom during the last extramarital sex. Low level of condom-use was attributed to condom not worthwhile (34.5%) and opinion (50.0%) condom not made for the elderly. Moreover, FGD participants viewed sex could not lead to pregnancy and majority (60.3%) posited patronizing traditional healers and few (10.3%) use of herbs/concussion could prevent HIV/AIDS. Similarly, non-condom use was due to confidence in traditional herbs, perceived to protect against STIs including HIV/AIDS.

## Conclusion

Engagement in risky activities among elderly is a growing HIV/AIDS challenge. Condom-use is misconstrued probably due to knowledge gap. Without urgent measures to enable them protect themselves, development efforts will be in jeopardy. Investing in geriatric SRH is cost-effective intervention in mitigating HIV/AIDS pandemic.

## Disclosure of interest

None declared

